# Ipsilateral diabetic striatopathy: A case of clinicoradiological discordance and evolving movement disorders

**DOI:** 10.5339/qmj.2025.60

**Published:** 2025-06-09

**Authors:** Subhankar Chatterjee, Payel Biswas, Samya Sengupta, Shambaditya Das, Ritwik Ghosh, Rana Bhattacharjee, Julián Benito-León, Souvik Dubey

**Affiliations:** 1 Department of Endocrinology & Metabolism, Medical College & Hospital, Kolkata, India; 2 Department of Radiology, GNRC Hospital, Barasat, Kolkata, India; 3 Department of Neuromedicine, Bangur Institute of Neurosciences, IPGMER & SSKM Hospital, Kolkata, India; 4 Department of General Medicine, Burdwan Medical College &Hospital, Burdwan, India; 5 Department of Neurology, 12 de Octubre University Hospital, Madrid, Spain; 6 Instituto de Investigación Sanitaria Hospital 12 de Octubre (imas12), Madrid, Spain; 7 Centro de Investigación Biomédica en Red Sobre Enfermedades Neurodegenerativas (CIBERNED), Madrid, Spain; 8 Department of Medicine, Faculty of Medicine, Complutense University, Madrid, Spain *Email: jbenitol67@gmail.com

**Keywords:** Diabetic striatopathy, diabetes, hyperglycemia, hemichorea, hemiballism

## Abstract

**Background:**

Diabetic striatopathy (DS) typically presents with hemichoreoballism and contralateral striatal lesions on neuroimaging. However, cases of unilateral movement disorders with predominant ipsilateral striatal lesions are rare.

**Case presentation:**

We present a case of DS in a 62-year-old woman from rural India with poorly controlled diabetes mellitus who developed acute-onset right hemichoreoballism. Neuroimaging revealed a predominantly right-sided striatal lesion, illustrating a clinicoradiological discordance—a mismatch between the clinical symptoms and radiological findings. Despite achieving tight glycemic control and administering neuroleptic medications, the involuntary movements demonstrated only partial improvement. Neurological changes persisted on the ipsilateral side of the affected limbs even after 1 year of follow-up. Notably, perioral dyskinesias developed during subsequent follow-up visits.

**Discussion:**

This report highlights the clinical and neuroradiological discordance observed in DS. The potential underlying mechanisms contributing to this paradox are explored and discussed.

**Conclusion:**

The clinical and radiological discordance in DS is a frequent yet under-reported phenomenon. However, the actual mechanistic underpinnings need to be addressed by advanced functional and structural neuroimaging.

## Introduction

Diabetic striatopathy (DS), an emerging complication of diabetic patients, is classically defined as the presence of acute onset of choreoballism and/or striatal hyperdensity on non-contrast computed tomography or striatal hyperintensity on T1-weighted magnetic resonance (MR) imaging in association with hyperglycemia.^1,2^ However, the spectrum of DS is expanding as varied reversible non-choreoballistic involuntary movements (either in conjunction with choreoballism or in isolation) are increasingly being reported in diabetic patients.^1,3^ Prompt diagnosis of DS is crucial, as it often leads to reduced management costs and excellent outcomes in most cases.^4^ However, up to 25% of patients may experience incomplete resolution, even with the use of additional anti-chorea or neuroleptic medications.^2,4^

DS can significantly affect patient-reported quality of life due to the debilitating movement disorders it induces, such as hemichorea and hemiballismus. These involuntary movements lead to functional impairments that disrupt daily activities and social interactions.^1,2^ Timely recognition and effective management, particularly through glycemic control, have the potential to substantially improve outcomes.^1,2^

Although hemichoreoballistic movements with contralateral striatal involvement in neuroimaging are the most common and classical representation of DS, clinicoradiological discordance has also been observed.^2,4^ We report a case of DS in a 62-year-old woman from rural India who was admitted to a government facility and presented with right-sided hemichoreoballism and predominant ipsilateral striatal involvement. This case highlights an unusual clinicoradiological discordance, characterized by a mismatch between clinical symptoms and radiological findings, along with a complex evolution of movement disorders over time.

Written informed consent was obtained from the patient’s daughter, ensuring a complete understanding of the case details and consent for its publication. As this is a case report, formal ethics committee approval was not required, in line with established guidelines.

## Case Presentation

A 62-year-old woman from India presented to the emergency room with acute-onset involuntary movements on the right side of her body that had persisted for 4 months. The movements were semiologically characterized as right hemichoreoballism, predominantly involving the lower limb (Video 1). She had a history of type 2 diabetes and arterial hypertension, diagnosed 3 years earlier after an acute coronary syndrome requiring angioplasty. Although her blood glucose levels were previously well-controlled with metformin (1 g once daily) and vildagliptin (50 mg once daily), she discontinued her medications 6 months prior to presentation.

Physical examination showed a body mass index of 18 kg/m^2^, a regular pulse of 80/min, and blood pressure of 130/80 mmHg. Apart from right hemichoreoballism, there was no other focal neurological deficit.

Initial investigations revealed a fasting plasma glucose of 550 mg/dL, postprandial plasma glucose of 699 mg/dL, HbA1c of 16.6% (National Glycohemoglobin Standardization Program), and serum creatinine of 1 mg/dL (estimated Glomerular Filtration Rate: 64 mL/min/1.73 m^2^). Additional tests, including arterial blood gas analysis, serum electrolytes, blood ketones, lipid profile, liver function tests, thyroid function tests, abdominal ultrasonography, and 2-D echocardiography, were all within normal limits.

**Figure 1:**
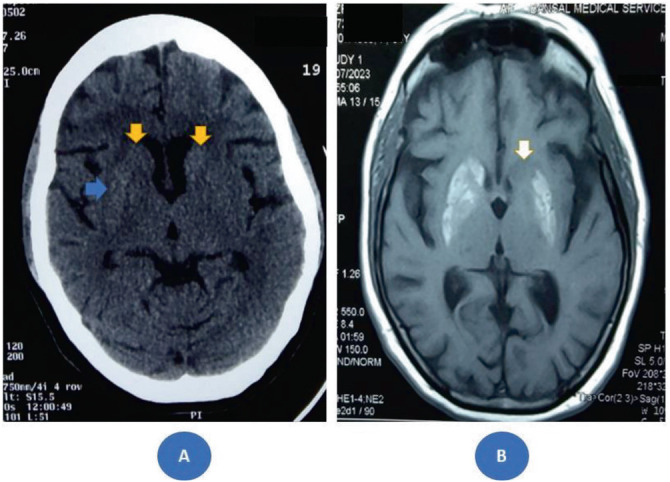
(A) Non-contrast computed tomography scan revealing right putaminal hyperdensity (blue arrow) and subtle bilateral hyperdensity in the head of the caudate nucleus (yellow arrow) at the time of initial presentation. (B) Magnetic resonance imaging displaying a sharply demarcated T1 hyperintense signal in the right striatum, right lentiform nucleus, left putamen, and left lentiform nucleus, with a normal appearance of the left caudate head (white arrow).

A computed tomography brain scan revealed subtle hyperdensities in the bilateral basal ganglia, with a greater radiological burden on the right side compared to the left (Figure 1A). T1-weighted MR imaging showed bilateral basal ganglia hyperintensity, more pronounced on the right side, with no evidence of hemorrhage or acute infarction (Figure 1B).

Based on the characteristic involuntary movements and T1 hyperintensity in the striatum associated with hyperglycemia, a diagnosis of DS was made, highlighting the significant clinicoradiological discordance.^1^

As the patient was unwilling to be admitted and refused insulin therapy, she was treated on an outpatient basis and followed up monthly. She was started on metformin 1 gm once daily, dapagliflozin 10 mg once daily, and teneligliptin 20 mg once daily. In order to control involuntary movements, tetrabenazine 25 mg twice daily, trihexyphenidyl 1 mg twice daily, and clonazepam 0.5 mg once daily were prescribed. For other comorbidities (ischemic heart disease and arterial hypertension), aspirin 75 mg once daily, atorvastatin 40 mg once daily, nitroglycerin controlled release 2.6 mg twice daily, telmisartan 40 mg once daily, and amlodipine 5 mg once daily were continued.

**Figure 2:**
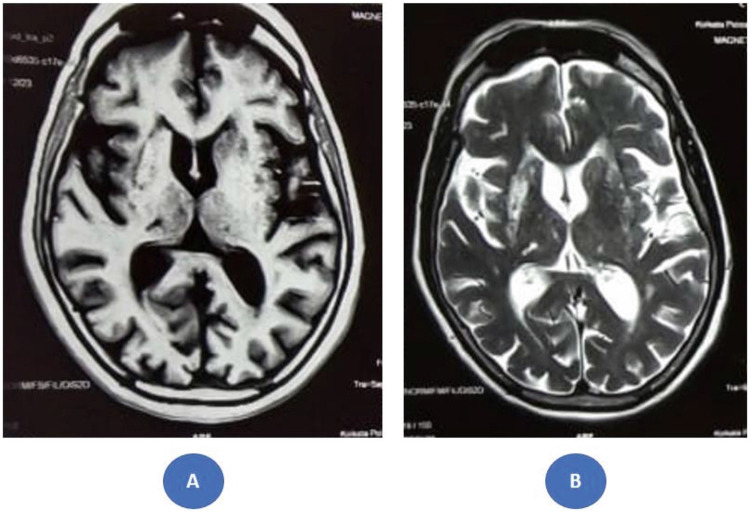
(A) Magnetic resonance imaging showing subtle T1 hyperintensity in the right striatum after 1 year of follow-up, with no appreciable signal alteration on the left side. (B) Corresponding T2 images displaying bilateral striatal hyperintensity (right > left) along with more pronounced putaminal atrophy on the right side.

After 3 months, despite achieving euglycemia (fasting plasma glucose: 84 mg/dL, postprandial plasma glucose: 125 mg/dL, and HbA1c: 5.4%) and the continued use of medications aimed at controlling the movement disorders, the abnormal movements persisted. While the involuntary movements in the right upper limb were nearly resolved, the choreoballistic movements in the right lower limb progressed to ballism. Notably, perioral dyskinesias emerged during this period. However, MR imaging demonstrated a significant reduction in the striatal disease burden (Figure 2). The patient has been followed in our neurology and diabetes clinics for the past year, with visits approximately every 3 months. Unfortunately, she continues to experience ballistic movements in the right lower limb, along with persistent perioral dyskinesias, both of which negatively affect her daily living and overall quality of life (Video 2).

Further detailed evaluation found no diabetic micro- or macroangiopathic complication apart from ischemic heart disease and grade 2 albuminuria (77.27 mg/gm).

## Discussion

This case is an excellent representation of clinicoradiological discordance or inconsistency in DS, a frequent yet under-reported phenomenon.^2,4^ Dubey et al.^5^ proposed a classification schema for DS: (A) Symptomatic DS, characterized by a typical clinical presentation with a corresponding striatal lesion on neuroimaging; (B) Clinically isolated DS, involving an acute-onset movement disorder with a temporal relationship to hyperglycemia but negative neuroimaging; and (C) Radiologically isolated DS, featuring characteristic radiological findings without associated movement disorders. Clinicoradiological discordance in symptomatic DS is defined as (a) a radiological lesion ipsilateral to the side of the movement disorder, (b) a unilateral radiological lesion with bilateral movement disorders, or (c) a bilateral radiological lesion with a unilateral movement disorder. Our case fits the last category, where the radiological burden was more pronounced on the ipsilateral side from disease onset.^1,5,6^ Interestingly, the radiological burden was significantly greater on the side ipsilateral to the clinically affected limbs from the onset of the disease. Even after a year of follow-up, some radiological signs persisted in the striatum ipsilateral to the affected limbs, while no residual DS-specific lesions were observed in the contralateral striatum on neuroimaging.

Lin,^7^ followed by Fong et al.,^8^ initially described cases of hemichoreoballism with ipsilateral putaminal involvement. The underlying cause of this clinicoradiological discordance remains unclear and seems to challenge the traditional lesion-manifestation theory of neurological localization.^5^ However, several hypotheses have been proposed. Given the burden of atherosclerotic vascular disease, it is possible that chronic lacunar infarcts already compromised the patient’s contralateral basal ganglia, and the non-ketotic hyperglycemic state unmasked the activity of these previously silent lesions.^7^ Fong et al.^8^ suggested that the ipsilateral striatal lesion might actually be silent, and the generation of movements was governed by the contralateral striatum, which harbored old lacunar infarcts. Another hypothesis, derived from stroke models, suggests that the non-decussation of corticospinal tracts to the contralateral side might explain this paradoxical event.^9^ Shunting of excitatory inputs from diseased, disinhibited ipsilateral basal ganglia to the contralateral motor cortex through corpus callosal connections could account for this rare phenomenon.^10^ Further support for the interconnectedness of basal ganglia and their control over bilateral motor cortices comes from observations that unilateral functional neurosurgery can result in bilateral improvements in motor function.^11–13^ However, more direct and objective evidence from advanced structural imaging techniques, such as diffusion tensor imaging, and functional imaging, like single-photon emission computed tomography, is needed to elucidate the anatomical and physiological mechanisms underlying this discordance in DS.^1,5^

Chua et al.^2^ reported that the median time for complete resolution of striatal changes on MR imaging was 6 months. In this case, while the contralateral striatum became radiologically disease-free, the ipsilateral striatum continued to show persistent, albeit fainter, signal intensity changes even after a year of follow-up. Notably, clinical improvement generally precedes radiological resolution.^2^ The persistence of some radiological burden in the ipsilateral striatum may contribute to incomplete recovery, as observed in our case, and this requires further investigation. There remains an open field of research to identify predictors of clinical outcomes in DS. Early radiological indicators, such as the initial severity of the radiological burden (preferably assessed by MR volumetric studies)^14^ and striatal atrophy and gliosis,^15,16^ may help predict partial or incomplete recovery or recurrence.

The perioral dyskinesias that became apparent during follow-up visits could be either a manifestation of DS^17^ or a side effect of neuroleptic medications used to treat the chorea.^18^ Further research is necessary to clarify these associations and to optimize treatment strategies.

## Conclusion

This case report highlights the occurrence of hemichoreoballism with an ipsilateral striatal lesion in DS, illustrating clinicoradiological discordance. Although this phenomenon is not uncommon in clinical practice, it remains under-reported and underappreciated. Advanced structural and functional neuroimaging research is needed to uncover the mechanisms behind this seemingly paradoxical presentation and to improve our understanding of DS.

Informed consent

Written informed consent was taken from the patient’s daughter to publish the videos, figures, and case details for educational purposes.

## Authors’ contribution

SC, RG, and SDubey generated the idea. SC, SS, SDas, SDubey, and RB were directly involved in the patient’s care and diagnosis. PB helped with the interpretation of neuroimages. SC wrote the first draft, which was further critically revised by PB, SS, SDas, RG, RB, JBL, and SDubey. All authors agreed upon the final version of the manuscript.

## Acknowledgments

We are indebted to Dr. Alak Pandit, MD, DM, Professor, Department of Neuromedicine, Bangur Institute of Neurosciences, IPGMER & SSKM Hospital, Kolkata, India, for his continuous support of our research endeavor.

## Conflicts of interest

Nil

## Funding

JBL is supported by the National Institutes of Health, Bethesda, MD, USA (NINDS #R01 NS39422), the European Commission (grant ICT-2011-287739, NeuroTREMOR), the Spanish Health Research Agency (grant FIS PI12/01602 and grant FIS PI16/00451) and The Recovery, Transformation, and Resilience Plan at the Ministry of Science and Innovation (grant TED2021-130174B-C33, NETremor).

## Figures and Tables

**Video 1:**
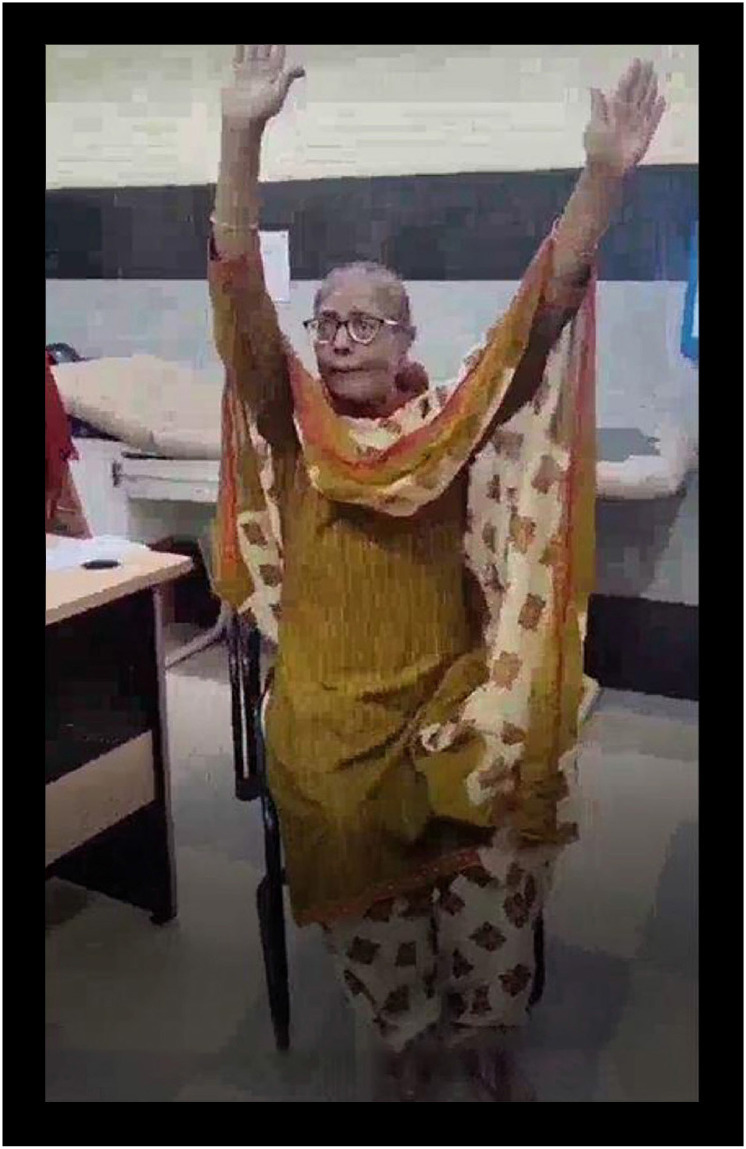
Right hemichoreoballism predominantly affecting the lower limb, observed during the initial visit (https://www.youtube.com/shorts/QOfJFimmqSk).

**Video 2:**
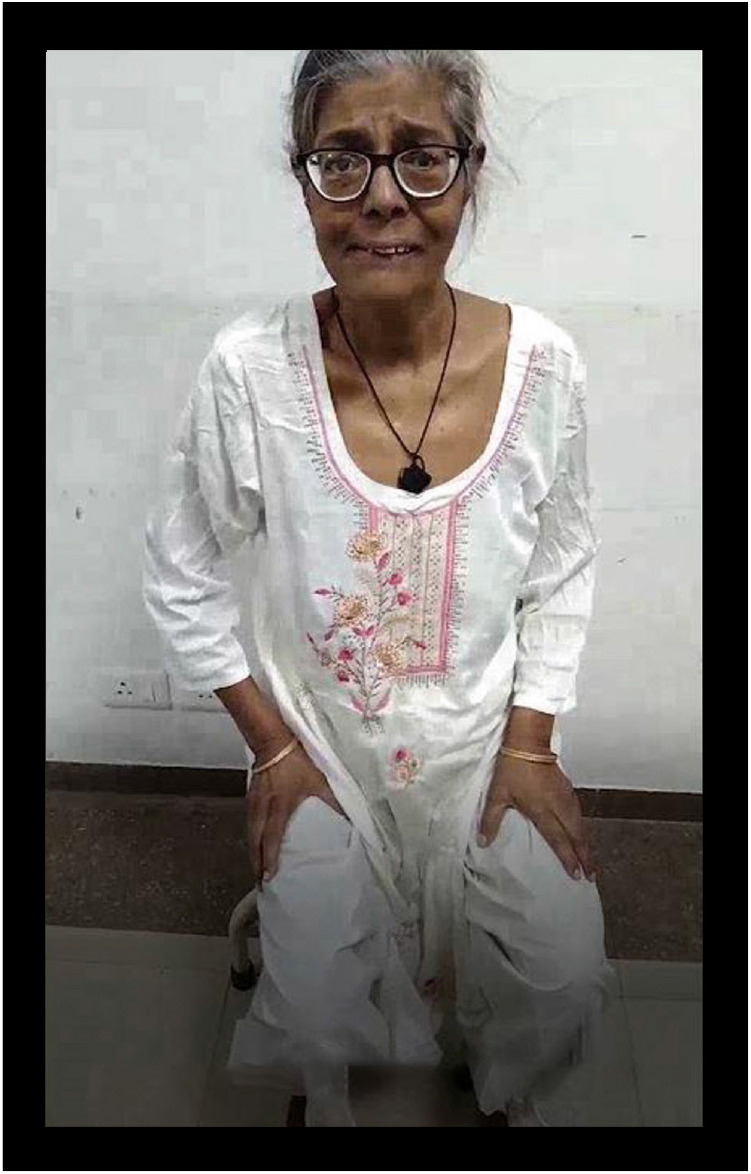
On follow-up, right upper limb movements had resolved. Choreoballism in the right lower limb evolved into monoballism, with the appearance of perioral dyskinesias and grimacing (https://www.youtube.com/shorts/eR_H0ARF5LY).

## References

[1] Chatterjee S, Ghosh R, Biswas P, Das S, Sengupta S, Dubey S (2024;). Diabetic striatopathy and other acute onset de novo movement disorders in hyperglycemia. Diabetes Metab Syndr.

[2] Chua CB, Sun CK, Hsu CW, Tai YC, Liang CY, Tsai IT (2020;). “Diabetic striatopathy”: clinical presentations, controversy, pathogenesis, treatments, and outcomes. Sci Rep.

[3] Mukherjee D, Chatterjee S, Sarkar P, Ghosh R, Das S, Ray BK (2023;). Expanding the spectrum of diabetic striatopathy: insights from a case of hyperglycemia-induced propriospinal myoclonus. Tremor Other Hyperkinet Mov (N Y).

[4] Dubey S, Chatterjee S, Ghosh R, Louis ED, Hazra A, Sengupta S (2022;). Acute onset movement disorders in diabetes mellitus: a clinical series of 59 patients. Eur J Neurol.

[5] Dubey S, Biswas P, Ghosh R, Chatterjee S, Kanti Ray B, Benito-León J (2022;). Neuroimaging of diabetic striatopathy: more questions than answers. Eur Neurol.

[6] Chatterjee S, Ghosh R, Dubey S, Feingold KR, Anawalt B, Blackman MR, Boyce A, Chrousos G, Corpas E, Endotext (2000). Diabetic striatopathy. In:.

[7] Lin JJ (2001;). Ipsilateral putamen hyperintensity on T1-weighted MRI in non-ketotic hyperglycemia with hemiballism-hemichorea: a case report. Parkinsonism Relat Disord.

[8] Fong SL, Tan AH, Lau KF, Ramli N, Lim SY (2019;). Hyperglycemia-associated hemichorea-hemiballismus with predominant ipsilateral putaminal abnormality on neuroimaging. J Mov Disord.

[9] Zhou LW, Chew J, Field TS (2021;). Teaching neuroimages: stroke with nondecussating corticospinal tracts causing ipsilateral weakness: straight forward. Neurology.

[10] Kannepalli NR, Yadav R, Vazhayil V, Somanna S, Pal PK (2016;). Ipsilateral hemichorea-hemiballism in a case of postoperative stroke. Tremor Other Hyperkinet Mov (N Y).

[11] Alberts JL, Hass CJ, Vitek JL, Okun MS (2008;). Are two leads always better than one: an emerging case for unilateral subthalamic deep brain stimulation in Parkinson’s disease. Exp Neurol.

[12] Slevin JT, Gerhardt GA, Smith CD, Gash DM, Kryscio R, Young B (2005;). Improvement of bilateral motor functions in patients with Parkinson disease through the unilateral intraputaminal infusion of glial cell line-derived neurotrophic factor. J Neurosurg.

[13] Shemisa K, Hass CJ, Foote KD, Okun MS, Wu SS, Jacobson CE 4th (2011;). Unilateral deep brain stimulation surgery in Parkinson’s disease improves ipsilateral symptoms regardless of laterality. Parkinsonism Relat Disord.

[14] Lin YT, Chen SC, Yip PK, Wang V (2019;). Magnetic resonance imaging volumetric analysis for diabetic striatopathy with two episodes of hemichorea-hemiballism syndrome: a case report. Medicine (Baltimore).

[15] Lucassen EB, Delfyett WT, Stahl MC (2017;). Persistent hemichorea and caudate atrophy in untreated diabetic striatopathy: a case report. Case Rep Neurol.

[16] Chatterjee S, Ghosh R, Ojha UK, Diksha, Biswas P, Benito-León J (2022;). Recurrent facial focal seizures with chronic striatopathy and caudate atrophy-A double whammy in an elderly woman with diabetes mellitus. Neurohospitalist.

[17] Arecco A, Ottaviani S, Boschetti M, Renzetti P, Marinelli L (2024;). Diabetic striatopathy: an updated overview of current knowledge and future perspectives. J Endocrinol Invest.

[18] Ganzini L, Heintz RT, Hoffman WF, Casey DE (1991;). The prevalence of tardive dyskinesia in neuroleptic-treated diabetics. A controlled study. Arch Gen Psychiatry.

